# A Tool for Reflecting on Questionable Numbers in Society

**DOI:** 10.1007/s11217-022-09836-6

**Published:** 2022-07-04

**Authors:** Kjellrun Hiis Hauge

**Affiliations:** Faculty of Education, Arts and Sports, Western Norway University of Applied Sciences, Bergen, Norway

**Keywords:** Critical mathematics education, Fake news, Deceptive numbers, Uncertainty, Critical citizenship

## Abstract

The increased distribution of fake news on internet and social media raises concerns for democratic processes. Sometimes, argumentation in deceptive information is built on numbers, which gives reason to include mathematics when working with fake news in education. In this paper, I suggest a tool to facilitate students’ critical thinking related to numbers, or other mathematical representations, presented in the media. It may not be straight forward, or even possible, to judge the validity of presented numbers, or whether numbers are used with the intention to deceive. Complex topics are associated with uncertainty, which implies that numbers may be questionable without hidden intentions, and that evaluating a number’s relevance may be quite challenging. The developed tool consists of a set of questions to help reflecting on the validity of numbers, which again is developed into categories reflecting degrees of validity and whether the mathematical representation has a deceptive role. The categories are illustrated with examples from the media and from a classroom situation in teacher education to indicate how the tool can help raise critical questions. The developed categories are based on academic literature on fake news, typographies of uncertainty and on critical mathematics education.

## Introduction

‘Fake news’ has become a frequent term in the media, and the phenomenon is a growing concern for democratic processes in society. Internet and social media enable to spread deceptive and manipulating news at a considerable speed and range. During the COVID-19 pandemic, internet has abounded with conspiracy theories and false information on advice, measures, cures and the spread of the virus.^1^ Fake news is often designed to trigger emotions at the expense of knowledge and facts, often with the intention to create fear (Gelfert 2018). Main drivers of such misinformation have been identified to be money and ideology (Rochlin 2017; Tandoc et al. 2018). Fake news often resembles real news, is based on anonymous sources and tend to consist of a mix of fabricated facts and acknowledged information (Berghel 2017). Validity may thus be challenging to evaluate, and not all of its content may deserve to be rejected.

Giroux (2018, p. 199) argued that a “democracy cannot exist without informed citizens and public spheres and educational apparatuses that uphold standards of truth, honesty, evidence, facts, and justice”. Fake news thus constitutes a threat to both individuals and to democracy, in particular when it becomes part of public debate or politics. Part of the academic literature on fake news differentiates between credible and non-credible websites (e.g. Jang et al. 2018). While becoming aware of non-credible websites and how fake news are distributed is essential, care should be taken in reducing the problem of fake news to make students aware of these websites or creating a dichotomy of true or false. On the contrary, it is imperative that students are aware that truth may not be accessible: Knowledge may be uncertain, biased or ambiguous.

There are several reasons for education to take a role in fighting implications of fake news. First, young people are considerable consumers of internet and social media, and studies show that young people may not be sufficiently capable in recognizing fake news (Notley and Dezuanni 2018). Second, education has a role within formation, as it prepares students for citizenship in present and adult life. Because fake news is considerably present in peoples’ lives, education should engage in problems of fake news (Peters 2018). Furthermore, education has a central role in politics because of its crucial role in shaping students’ values and identity together with developing their consciousness and agency, which is particularly important when facing fake news (Giroux 2018). A third reason for education to take responsibility is that schools (and universities) may offer a safe arena where students can practice facing disagreements. While people choose friends with more or less the same worldview, and many occupations attract employees who resemble each other (similar education, similar values, similar experiences), school classes often consist of students with various backgrounds, lifestyles and political stances. Because school classes do not need to take significant decisions, the classroom can be a safe arena for presenting different experiences and facing disagreement (Iversen 2014). Education institutions thus have an opportunity to engage students in discussions where they can explore disagreements in respectful ways. Students who get acquaintance with opposing argumentation and get used to disagreement have shown to be of great value, as they become more interested in politics and are more prone to start discussions with others who disagree (Hess and McAvoy 2015). Discussing controversial issues, of which many are accompanied with fake news, in class can provide a buffer against the worldwide increasing polarization in politics and social media, caused by the so-called echo chamber and by harsh attacks on people with other opinions.

A thorough elaboration on what it requires to develop students’ critical thinking related to a post-truth world is presented by Chinn et al. (2021). They provide five recommendations for curriculum development: (1) to design authentic learning environments, (2) present cases where knowledge is limited, and in different ways, (3) explore different ways of knowing, (4) promote constructive virtues and ideals connected with evaluating information and (5) promote understanding of how various institutions gain and present information. The recommendations acknowledge that critical thinking is essential, in particular related to different knowledge sources having different limitations due to how data and information is gathered. This also concerns knowledge represented by numbers or other mathematical representations, but numbers are not specifically addressed in the paper.

Fake news may include numbers, or other mathematical representations such as graphs or ratio images, so that inspecting numbers and reflecting on their role is relevant for critical citizenship. Numbers are often seen as objective and necessary in order to provide just measures in society, but they may also hide assumptions that cause unintentional injustice (Porter 1996). Mehta and Guzmán (2018) illustrated several ways to deceit an audience with numbers in multimodal news designs without bluntly lying, and Huff’s (1991) classic book shows a variety of ways statistics can be misused or deceptive. This means that numbers can be an effective manipulative tool without being fabricated.

Academic literature on statistical literacy has long promoted the value of developing students’ ability to scrutinise statistics presented in society (e.g., Watson 1997; Gal 2002; Weiland 2017). Moreover, a significant part of critical mathematics education is devoted to students’ critical thinking and critical citizenship as key means and purposes (e.g. Skovsmose 1994, 2011; Gutstein 2013). For example, Skovsmose (1994) has been concerned with how mathematics based criteria colonise decision-making in society and influence how we understand and shape reality. He has denoted this as the *formatting power of mathematics*. Gutstein (2003) developed the term *reading and writing the world with mathematics*, expressing how the world can be described and understood through mathematics and how action can be shaped through mathematics. He leaned on Freire’s ideas on literacy and applied these terms when describing how social justice can be addressed in mathematics classrooms and how mathematics can empower students. Further, education literature on mathematical modelling (representing a problem through mathematical relations and expressions) and statistics provide applications of a range of societal topics, demonstrating that mathematics is about more than correct and wrong answers, but that mathematics in society is normally uncertain (e.g. Weiland 2017).

The above examples support the idea that mathematics education can play a role in enhancing students’ democratic potential. Teaching and learning will then concentrate on engaging students *with* and *through* mathematics rather than *in* mathematics, focussing on how mathematics works in society (Stemhagen and Henney 2021). The idea breaks with absolutist perspectives on mathematics education, as mathematics applied in society is rarely about objectivity and right or wrong, and it breaks with constructivist perspectives, as these still aim at getting right answers, although the focus is on conceptual understandings that make sense to the individual student. Democracy oriented mathematics education, on the other hand, is about purpose and context of mathematics, and align with students’ early beliefs about what mathematics is, because mathematics education at early stages normally introduces concepts through practical examples (Stemhagen and Henney 2021). Emphasizing purpose and context is highly relevant for fostering students’ critical thinking related to numbers presented in fake news.

Addressing fake news in mathematics education is a research area at an early stage. Kollosche (2020) argued that mathematics education is not prepared for post-factual times because of contradictory and unhelpful epistemological positions on the notion of truth. One position, he pointed out, is mathematics having the role of proving whether mathematical representations are misused or misinterpreted. This resembles the idea described above, that fake news can be revealed through checklists, indicating an absolutist orientation based on a true—false dichotomy. Another truth position is a post-modern position where truth is relative. Kollosche argued that these epistemological positions are counterproductive in fighting fake news. Instead, he suggested to focus on styles of reasoning that are agreed to facilitate factual discourses, among others, differentiating between statistical reasoning and reasoning based on postulations. These styles are ideal styles of reasoning, while, as he draws attention to, mathematical models are arbitrary to various extents. Educating students in these styles may be very helpful in supporting understandings of factual reasoning, but because they are ideals, awareness is also needed on how applied statistics and mathematical models may deviate from ideal situations and that choices in problem framing and what data to collect may influence the outcome of a particular reasoning. This applies especially related to topics of politicised applied mathematics and social justice, and can be associated with Skovsmose’s (2020) call for a social and ethical dimension of reflection on mathematics in mathematics education. In practice, styles of reasoning need to be accompanied by social and ethical reflections.

In a previous paper, I (Hauge 2019) argued that working with fake news in mathematics classrooms will require investigating the context of presented numbers, possible manipulative aspects and reflecting on whether numbers may be fake or just uncertain. In line with these ideas, colleagues and I (Hauge et al. 2019) investigated numbers presented in a Youtube conspiracy theory. Both papers presuppose that factual reasoning is possible, but that reasoning may be conclusive to various degrees when applied on societal problems. Numbers in the media are often presented without error bars or statements on limitations, which means that numbers from different sources that do not match may incorrectly be refuted as wrong. It may be challenging to conclude whether a number is false or not and whether there is a deceptive intention behind a number.

I (Hauge 2019) applied the characteristics *uncertain, wrong, twisted and fabricated numbers* when reflecting on numbers, but the characteristics were not developed, further discussed or justified. Because it is essential to critically reflect on the role and validity of numbers presented in the media, it is the purpose of this paper to develop a tool with useful ideas and concepts. The aim is thus to provide a framework with categories of the validity of numbers and whether there is an intention or not to misguide through numbers or other mathematical representations. The framework also includes questions to help reflect on these perspectives. The categories are developed based on literature on fake news, uncertainty typologies and critical mathematics education. The tool is illustrated with examples from real life.

The academic discipline of statistics is developed to handle quantified information and to tame or control uncertainty through estimating uncertainty, for instance represented by standard deviation or probability. In the following, I present literature on how statistics can be misused and literature on uncertainty which statistics cannot estimate in a sufficient and relevant manner. Both misuse and insufficiency fail to present drawbacks of quantified information, but differ in intention.

## Manipulation with Numbers

Spotting and categorising deceptive quantities in the media has been addressed in different ways. Kress (2004) illustrated how images representing quantities open up for various interpretations compared to written texts. This openness requires an awareness of what the essential message is to be communicated when choosing the image design, as images can be deceptive, also when representing quantities. Deceptive quantities were the focus of Mehta and Guzmán’s (2018) analysis of multimodal media outlets during the U.S. presidential elections in 2016. They looked for visual, spatial, and textual manipulations and showed how numbers and mathematical representations can have a manipulative effect while not “bluntly lying”, as they expressed it. Four deceptive design categories were identified: (1) *Spatial manipulation and biased design* represented cases where significant information was left out so that the mathematical representation gave a biased impression of the story; (2) *fantasizing with probability* signified how choices of mathematics related terms, such as *probability*, has the potential to be misunderstood if the term is not adequately understood in the given context. In addition, great numbers written numerically may stand out visually in texts and can catch attention, but may be deceptive if the associated text is not read thoroughly; (3) *manipulation through data extrapolation* denoted cases where an insignificant or local problem was generalised in time or space without a rationale for it; (4) *avoiding numbers when inconvenient* covered situations where words were expressed instead of numbers, such as, *many* without suggesting how many, giving the impression that a claim had sufficient support. Taken together, quantitative and mathematical representations appear convincing so that in combination with emotional effect, multimodal news can be quite deceptive.

Tufte (2001) has given examples on how graphs have been deceptive in newspapers, and which can be placed in Mehta and Guzman’s first category. They include: showing only part of the data to convey an exaggerated trend while neglecting the rest; showing a graph where the columns do not represent comparable units; visual trickeries; disappearing baselines (the y-axis does not start at zero); biased representation due to missing factors; design variation (mixing designs in a graphics so that comparing elements is confusing), and; making exaggerated differences when illustrating a change through areas or volumes (for example illustrating an actual number increase of order 2 through pictures of a volume where the illustrated increase is in all three directions, resulting in an increase of 2 × 2 × 2). Such trickeries and biases can be deceptive, even when the actual numbers are presented in the graph.

The categories and examples presented in this section are useful to be aware of when considering the validity and role of numbers. To strengthen the capacity to reveal such trickeries and biases, students need to be presented by authentic stories from the media, in line with the first recommendation by Chinn et al. (2021) (see above). Inspecting biases in stories is crucial for understanding limitations of presented knowledge (recommendation 3 by Chinn et al.). However, biases due to missing factors can be far more challenging to reveal. This is addressed in the following where limitations of knowledge is connected to uncertainty.

## Categories of Uncertainty

Numbers can be uncertain without being flawed or manipulated, but uncertainty can still give reason to scrutinise numbers for their validity and relevance. Funtowicz and Ravetz (1990) argued that qualitative aspects of uncertainty are essential to assess and communicate when quantified information is applied in society. They differentiated between three sorts of uncertainties: (1) *Inexactness,* which can be expressed with statistical measures (standard deviation, probabilities, etc.); (2) *Unreliability*, which is a consequence of simplifications and other choices in knowledge production. For example, there may be knowledge about how people become infected by the corona virus, but translating this to a mathematical model on distancing requires generalisations and simplifications. The resulting uncertainty will be beyond inexactness because all uncertainty will not be captured in formalised methods. The final sort of uncertainty, they called (3) *border with ignorance*, which denotes lack of knowledge, data, methods or consciousness of aspects of a problem, for instance how the global economy will change as a consequence of the pandemic. Such uncertainty is common in complex problems.

While inexactness can be properly represented through statistical measures, neither unreliability nor border with ignorance can be sufficiently quantified. There is no clear distinction between the sorts of uncertainty in practice, because the uncertainty depends on the stakes of the issue at hand. For example, unknown consequences become more significant in decision making when the involved parties may be affected in different ways, depending on how the uncertainty is handled.

Wynne (1992) developed a similar set of uncertainty categories to the one by Funtowicz and Ravetz (1990), but added a fourth category *Indeterminacy*, which covers uncertainty characterised by the lack of possibility to foresee how people, companies or institutions will act in situations, but may contribute to making risk consequences indeterminate. For example, it is unknown what new risks will appear as a consequence of individual and political decisions on the COVID-19 pandemic. Similarly, van der Sluijs (2017) operated with the uncertainty category *limited social robustness*, which connects coping with uncertainty to societal relevance.

Several assessment frameworks have been developed based on Funtowicz and Ravetz’ (1990) three sorts of uncertainty by incorporating relating aspects. The frameworks have in common that they assess uncertainty in science-based advice, where quality in advice is seen in relation to context, values and qualitative aspects of uncertainties (e.g. Walker et al. 2003; van der Sluijs et al. 2008). The frameworks are positioned in the philosophy of post-normal science, founded by Funtowicz and Ravetz (1990), who argued that quality in science for policy in cases where knowledge is uncertain, stakes are high, values in dispute and decisions are urgent, should be understood in relation to the decision at hand. First, assessing and communicating uncertainty is key to understanding strengths and drawbacks of science-based decisions. Second, Funtowicz and Ravetz (1990, 1993) called for an extended peer review to evaluate the relevance of information. They suggested a democratic form of evaluation, where peers from different interest groups and lay persons were invited to ensure multiplicity of perspectives.

Post-normal science has been criticised for these perspectives on quality. Peters (2018), for example, worried that extended peer reviews will give more acceptance to false facts and fake news. Saltelli and Funtowicz’ (2017) on the other hand, argued that extended peer reviews can help regain trust in science, after extensive experiences with deceitful nutrition advice from doctors and economists’ exaggerated confidence before the finance crisis. In addition, complex issues, such as the COVID-19 pandemic, are often associated with a range of known unknowns which experts and decision-makers cannot efficiently handle alone (Waltner-Toews et al. 2020). These are reasons to include lay persons in decision making on complex problems. This is challenging and requires that experts and decision makers convey uncertainty in a rich and open manner, as suggested above. In addition, it is crucial that lay persons (e.g. students) learn limitations of knowledge from various research areas, in line with recommendations by Chinn et al. (2021).

## Uncertainty Categories in Mathematics Education

Uncertainty perspectives from post-normal science and from Wynne have also been embraced by scholars in mathematics and science education (see Barwell 2013; Wals 2012; Collucci-Gray et al. 2005; Christensen 2009; Kolstø and Hauge 2019; Hauge and Barwell 2017). The categories have been found relevant as they bring attention to, and awareness of, roles of quantifications in society and limitations of scientific and mathematized knowledge, qualities that are essential for reflecting on the validity of information. I have previously analysed how pre-service teachers reflected on uncertainty in climate change predictions, where Funtowicz and Ravetz’ uncertainty categories were applied, but merged into two: controllable and non-controllable uncertainty (Hauge 2016). The idea was to simplify the above uncertainty typologies, but still distinguish between uncertainties that can be sufficiently represented by statistical measures (controllable or tamed uncertainty) and those who are not. Attention to qualitative uncertainty characteristics is relevant for democratic engagement and possible critique to how experts and society handle uncertainty. Including such perspectives in education is in line with Weiland’s (2017) call for the academic field of statistical literacy to include critical reflection in investigative practices in statistics education in addition to understanding critical as evaluative.

An essential part of critical mathematics education is devoted to being an arena for developing critical citizenship (see e.g. Skovsmose 1994; Sánchez Aguilar and Molina Zavaleta 2012; Weiland 2017). Mathematics education often operates with problems with a single correct answer, while real-life problems seldom are associated with a single best solution. This implies that critical thinking related to applied mathematics in society and the role of mathematising in society is a crucial mathematical skill for critical citizenship. Barwell (2013) and Hauge and Barwell (2017) found that the notion of critical citizenship in critical mathematics education was comparable to the idea of extended peer communities in post-normal science. Exploring media stories to reflect on the validity of presented numbers and other content is thereby relevant for students’ critical citizenship.

Mathematising a real-world problem normally requires making choices regarding assumptions, simplifications and constraints. Similarly to the field of post-normal science, (and to part of science, technology and society (STS) studies), research literature within statistics and critical mathematics education is concerned with uncertainty associated with such choices. Small differences in translating the problem at hand to a mathematical model may affect the model outcome. This is a concern that calls for an extended peer review, according to post-normal science, and likewise, critical citizenship in critical mathematics education (Hauge and Barwell 2017).

Validating and critiquing mathematical models in education can be useful for developing students’ insights in limitations of knowledge, in line with the third recommendation by Chinn et al. (2021) when preparing students for facing fake news. Uncertainty can be addressed through validating the quality of data, the relevance of assumptions and evaluating the model output, comparing the model with alternative models, considering the effect an altered assumption would have for the model output, and in general, reflecting on consequences of applying the model in real life (Niss 2015, pp. 78–79). Such validation addresses uncertainty that may not be sufficiently represented through statistical measures, i.e. uncertainty which does not fall within Funtowicz and Ravetz’ category of *inexactness*. This implies that uncertainty explored through reflecting on a model’s various impacts on decisions is associated with the categories *unreliability, border with ignorance* and *indeterminacy,* described above.

Validating numbers may be challenging of several reasons: numbers can be fabricated, but challenging to check; the issue may be too complex to evaluate; intentions may be impossible to reveal, and; numbers may plainly be uncertain and not wrong (Hauge et al. 2019). Even a simple mathematical act, such as counting, can be associated with uncertainty. An incident in a kindergarten illustrates this point: When asking small children to count the number of beans in a pile, a child stated that there were also beans in the pile that were only pieces (Andersson and Wagner 2018). Should they be counted? This exemplifies how context is significant, and that even counting may not provide one single correct answer. The authors used the example to illustrate micro-politics of counting, problematizing political processes of deciding what to (not) count, among others (Andersson and Wagner 2018). How numbers are used may also be significant for the understanding of an issue. Numbers can provide clarification and may even express a form of respect, for example to the suffering of indigenous children when Canada was colonised (Andersson and Wagner 2017). Similarly to what Mehta and Guzmán (2018) addressed (see above), Andersson and Wagner questioned the use of vague wordings in earlier reports and asked whether this was to mask sufferings.

Skovsmose’s (1994) concept of *the formatting power of mathematics* is central when considering power perspectives of mathematised information. He described it as how mathematics influences our understanding of reality, for instance how mathematics shapes our lives through its central role in technology development, or how mathematics based systems shape our understanding of what is a fair distribution of goods in society. Power perspectives may often be linked to aspects of uncertainty. Child support, for instance, can be calculated by a range of different principles, which means that there is no single correct answer to what is a just distribution. The example illustrates that results from mathematising a problem may vary, depending on choices. This is related to what Walker et al. (2003) call uncertainty in the problem framing and concerns all mathematical modelling where the model is a simplification of a real-world problem and the output of the model influences some kind of action in society. Inspecting the problem framing is relevant when considering whether numbers are manipulated, in line with two of Mehta and Guzmán’s (2018) categories: (1) Spatial manipulation and biased design and (3) manipulation through data extrapolation, as they both include deliberate choices to ascertain a predefined result.

Taken together, there are several capacities useful for validating a number in a certain context: Firstly: grasping statistical concepts of uncertainty, such as probability and standard deviation, secondly: an understanding that there are situations where statistical measures may not capture essential uncertainties, and thirdly: an awareness of how choices in mathematising a societal question may influence the results and thereby have different effect on decisions.

## Questions to Reflect on the Validity of Numbers

I now extract points from the discussions above to suggest questions for reflecting on the validity of numbers or other mathematical representations. The purpose is to provide a tool for facilitating critical thinking related to numbers presented in the media, reports etc., where they have a role in informing on social and political topics. Numbers are rarely unambiguously correct or completely wrong. Rather, as shown above, they show part of a picture. In reflecting on numbers in the media, I argue that it requires exploring the numbers’ context, associated uncertainty, their source, communication forms but also own reflexivity (see also Table 1 for suggestions on questions).

**Table 1:** Suggested set of questions to help explore the validity of numbers

The number’s context	What is the purpose of the story?
	What do the numbers illustrate?
	What is the role of the numbers?
Communication forms	What communication techniques are applied?
	What emotions does the story appeal to?
	What group of readers does the story turn to?
Associated uncertainty	How is uncertainty and the surrounding complexity of the issue expressed?
	Is limitations of data, methods and results described?
	What is the degree of consensus?
Sources	What is the source of information?
	What is known about how the source gathers information?
	Are there other sources of information to compare with?
Reflexivity	What are my reasons to reason the way I do?
	What strengths and limitations do I have to reason soundly?
	How may my preferences and emotions affect what or whom I trust?

The numbers’ context is crucial to grasp when considering the validity of presented numbers, first of all the purpose of the story. For example, may the intention be merely to inform or rather to convince the reader that a specific course of action is necessary? And what role do the numbers play in this context? What do they serve to achieve? Such questions prepare for a discussion on intentions and the formatting power of the presented numbers (see above text on Skovsmose 1994). The communication form of the story is another aspect to inspect, what techniques are applied to appear convincing. Perhaps the numbers have the role to appear objective (in line with Porter 1996), or the numbers are accompanied with techniques to spark emotions, which is common in fake news (Gelfert 2018). Further, it is essential to reflect on the audience: who does the text turn to? Such questions bring attention to intentions and can be helpful in considering whether numbers may be part of a deception.

When investigating numbers, a crucial aspect to consider is associated uncertainties. Is uncertainty addressed at all? What about assumptions and limitations of computations or statistics? The more complicated or complex a topic or problem is, the more unlikely unequivocal answers are, and the more unlikely that statistical measures capture uncertainty sufficiently, so that uncertainties can be categorised as unreliability, uncontrollable, ignorance or indeterminacy (see section above). To reveal uncertainty, relevant questions could be: What connected topics or sub-topics might there be? Are essential aspects of the topic left out? What unknown aspects may there be? Are there conflicting views about the topic? Are single best answers possible? Are the numbers relevant in spite of being uncertain? These questions are related to the recommendations by Chinn et al. (2021); to let students inspect cases where knowledge is limited and to get acquainted with limitations of various forms of knowledge produced by different institutions etc., although they do not address quantified information specifically. This is also connected to the source of information, so that inspecting the source, how the source gathers information and achieves knowledge is part of getting a picture of uncertainties. A helpful way to reflect on the trustworthiness of numbers is to check whether there is consensus on the validity of numbers and information by searching for alternative sources and comparing numbers and conclusions. If these differ, exploring the complexity of the issue may be useful because different approaches to describing complex issues may produce different numbers without being tampered with. For example, topics within climate change and immigration are often complex. While lack of consensus on numbers may imply that a source is not trustworthy, it may also be due to disagreement between experts. For example, lack of consensus between scientists do occur when a research area is new and developing or when the complexity of the problem allows for different approaches and results (Funtowicz and Ravetz 1990).

Because most people tend to believe in information that aligns with their beliefs, some reflexivity related to investigating own beliefs and reasoning may be useful. This concerns both when news or information supports and contradicts own beliefs. Questioning own reasoning, preferences and capacity to make conclusions may raise awareness of the soundness, (or lack of soundness), of judging the validity of numbers. Being reflexive can be regarded as an essential virtue in education for a post-truth world, adding to the virtues highlighted by Chinn et al. (2021), such as being open-minded, fair-minded and intellectual virtues.

The validity of presented numbers (or other mathematical representations) can be discussed based on questions similar to the ones suggested above and in Table 1. Because questionable numbers are not necessarily part of fake news, but may be a result of a problem too complex to produce relevant numbers, I sketch a table (Table 2) where numbers ideally can be categorised according to whether a possible deficiency is intended or not, in addition to the numbers’ assessed validity. One dimension, the rows of Table 2, is thus labelled as i*ntended* and *unintended* in the rows of Table 2, while the other dimension is the validity of a number, or the degree of questionability. These categories are systemised in Table 2 on a scale from *invalid* numbers, the greatest degree of flaw, through *highly questionable* and *partly questionable* until *valid numbers*. I emphasise that that this scale is not meant to be linear. Neither is it likely that numbers can be objectively evaluated as to belong in a certain category. Rather, Table 2, together with questions such as in Table 1, is meant to be a thinking tool for reflecting on the validity of presented numbers and whether numbers have a manipulative role. Questions in Table 1 can help arguing why numbers may belong to one category rather than another.

**Table 2:** Categories of numbers or other mathematical representations based on the degree of deficiency and whether the deficiency is intended or not

	Invalid	Highly questionable	Partly questionable	Valid
Intended	Fabricated	Distorted	Tweaked	Valid
Unintended	Mistake	Ignorance	Biased	Valid

From posing questions suggested in Table 1, numbers may be concluded or suspected to be made up and wrong by intention to serve a certain purpose. Such numbers are here categorised as *fabricated*. In this case, numbers are just made up to make a convincing argument or as part of a clickbait (in line with Rochlin 2017). Numbers can also be wrong unintentionally, by accident. Such numbers are here categorised as *mistakes*. Mistakes happen when for example there is a misunderstanding, when wrong data are fed into a computer program or a computation is wrong by accident. However, in order for numbers to be invalid, the mistake must be significant.

The next categories are denoted as *highly questionable numbers*, where the numbers are not very relevant for the topic of concern. Numbers are noted as *distorted* if they are largely manipulated by purpose, for example taken out of context so that they are not representative, which correspond to the grave examples addressed by Mehta and Guzmán (2018) and Kress (2004) when presenting their manipulation categories. Although the idea is that numbers placed in the category of *distorted numbers* are not fabricated, the distinction between distorted and fabricated may not be sharp. For example, a number may not be fabricated in the sense that it is all made up, but may be chosen somewhat higher or lower than some source. This way, the number can be understood as partly fabricated.

Numbers can also be quite way off without any intention to be so, for instance when there is lack of knowledge, misunderstandings or lack of perspectives included in calculations. The result may be highly questionable numbers, i.e. quite irrelevant for the problem at hand or uncertain whether they are relevant. This is a situation associated with uncertainty in line with ignorance and indeterminacy in Wynne’s (1992) and Funtowicz and Ravetz’ (1993) uncertainty typographies.

The categories within *partly questionable numbers* are intended to cover less questionable numbers than the former categories. The difference is that they may be relevant and valuable to some extent, but may still be quite off so that how valid they are in a presented context can still be discussed. If numbers or the context of a number is manipulated, but relevant to some degree compared to distorted numbers, they are here denoted as *tweaked*. In political debates, it is common that numbers are used to pinpoint an argument and that politicians from opposing parties operate with slightly different numbers. Numbers can be tweaked in the sense that they do not show the whole picture, but carefully chosen to favour a certain view and associated solution, in line with Mehta and Guzmán (2018), Tufte (2001) and Kress’ (2004) manipulation categories.

The equivalent case *for partly questionable numbers* that are not manipulated, are here denoted as *biased numbers*. Bias is a statistical concept, representing a systematic difference between an estimated parameter and the true parameter. In this paper, the category *biased numbers* will go beyond this understanding to also include an everyday meaning of bias. This may be in situations where data or information are not sufficient to guarantee the correctness or relevance of a number representation. It has for example been claimed that earlier estimates of the pace of ice cap melting have been too low, and that involved experts have been too cautious in their conclusions, in fear of being criticised by climate sceptics for exaggerating consequences (Oppenheimer et al. 2019). The resulting numbers could be characterised as biased, however, the problem of ice cap melting and its consequences is complex, so that it may also be associated with ignorance, depending on how the consequences of the uncertainty is perceived. Misunderstandings may also fall under this category, for instance when President of France Macron expressed his concerns about the fires in the Amazon rain forests in 2019. He claimed that the Amazon produces 20% of the Earth’s oxygen, something that was refuted by experts. Zimmer (2019) indicated that he may have confused numbers, which suggests that Macron’s intention was not to misguide.

The purpose of dividing questionable numbers into highly and partly questionable numbers is that while partly questionable numbers may be partly relevant, highly questionable numbers may not be valid. On the opposite side of the scale from invalid numbers is the category *valid numbers*. It makes little sense to categorise a number as unintentionally valid. If a number is valid and unequivocally represents the problem at hand, there is hardly any room for manipulation. This implies that *valid numbers* is not differentiated like the other category headings. Uncertain numbers are here chosen to be valid numbers if the uncertainty is sufficiently represented through statistical measures. This corresponds to Funtowicz and Ravetz’ (1990) uncertainty category *inexactness*.

As will be illustrated, there are no rigid borders between the categories. They rather flow into each other since determining someone’s intention may be impossible, and since there is no objective way of concluding on the degree of deficiency or qualitative aspects of uncertainty. Rather, the categories represent different characteristics of numbers in contexts, which are fruitful to reflect on when exploring or critiquing numbers and their role, whether the topic is fake news or a political debate. Questions in Table 1 can help reflecting on how possible it is to actually categorise the numbers at hand. In the following I illustrate the categories with examples and reflections.

## Examples of Application

Colleagues and I (Hauge et al. 2019) explored the validity of numbers presented in a video which aimed to convince watchers that the world will be conquered by Muslims. The video operates with fertility rates, of which several deviated somewhat from official statistics, suggesting a lack of consensus. We were not able to conclude whether the numbers were fabricated because the source was unclear and there are several ways to calculate fertility rates and which produce different numbers. However, the deviating fertility rates in the video gave more support to the video message compared to the official rates. We (Hauge et al. 2019) concluded that some of the presented numbers were, what we called, twisted, because unstated underlying assumptions were highly questionable, numbers were taken out of context, numbers might have been adjusted, numbers were used with the intention to support a certain message and the numbers had a manipulative role. We also found that the origin of these numbers were not specified, contributing to a lack of transparency. Regarding each deviating number separately would suggest that the category *tweaked* in Table 2 would fit well. However, regarding the whole video altogether, with all its numbers and how they are used, suggests that the category *distorted numbers* would be more suitable. This means that the full picture and the context of the numbers may play a central role in considering consensus on the content of the video, the extent of manipulation and how numbers can be categorised. Taken together, questions on the numbers’ context, communication forms, associated uncertainties and sources (see Table 1) were relevant in reflecting on the numbers’ validity. Considering the reflexive questions in Table 1, it should be noted that I, as most researchers, have trust in official statistics, and I do not believe in this kind of conspiracy theories. I belong to an elite in society, and I am aware that others have less trust in official statistics who would conclude otherwise. Students with other beliefs need to be handled with respect in classrooms, but how is outside the scope of this paper.

A range of misinformation has been distributed in the media in the wake of the COVID-19 pandemic: conspiracy theories on how it emerged, suspicious medical advice and questionable claims on what measures are reasonable for society in fighting the virus.^2^ A rather common argument against lockdown early in the pandemic, was that COVID-19 is comparable to the flu in terms of how lethal it is. Such claims have reference to numbers: the number of infected, number of deaths and the now well-known reproduction number R—the average number of people that one infected person will pass the virus on to. Consensus on such numbers is not always the case, as numbers are associated with uncertainty due to the number of tested people, quality of tests, demographic variety, reporting principles, political measures to prevent spread, quality of health care and other factors. This means that it may be impossible to find representative statistical measures of associated uncertainties, which implies that the numbers are likely to be questionable to various extents, although conclusions have become more sound over time with more data and experience. Uncertainty regarding official numbers might thus be characterised as *ignorance* in the beginning of the pandemic, but moving to *bias* after more knowledge has been gained. Because misinformation can be a consequence of not knowing (for instance if similar numbers on the flu are not available in order to make comparisons between the two diseases), misinformation can be a result of either not knowing (*ignorance* or *bias*) or misleading with intention (*distorted* or *tweaked numbers*).

Questions on uncertainties together with their sources (see Table 1.) can facilitate critical reflections on the role of presented numbers on the COVID-19 pandemic in society. The reproduction number, spread and mortality rate are based on rather simple mathematical models, which can be explored through changing assumptions and conditions: What happens with the spread/mortality rate if….? What consequences would this have for society? Who will benefit from what kind of resulting measures? The pandemic thus illustrates the importance of considering the context of numbers and how society responds to uncertainty.

**Fig. 1:**
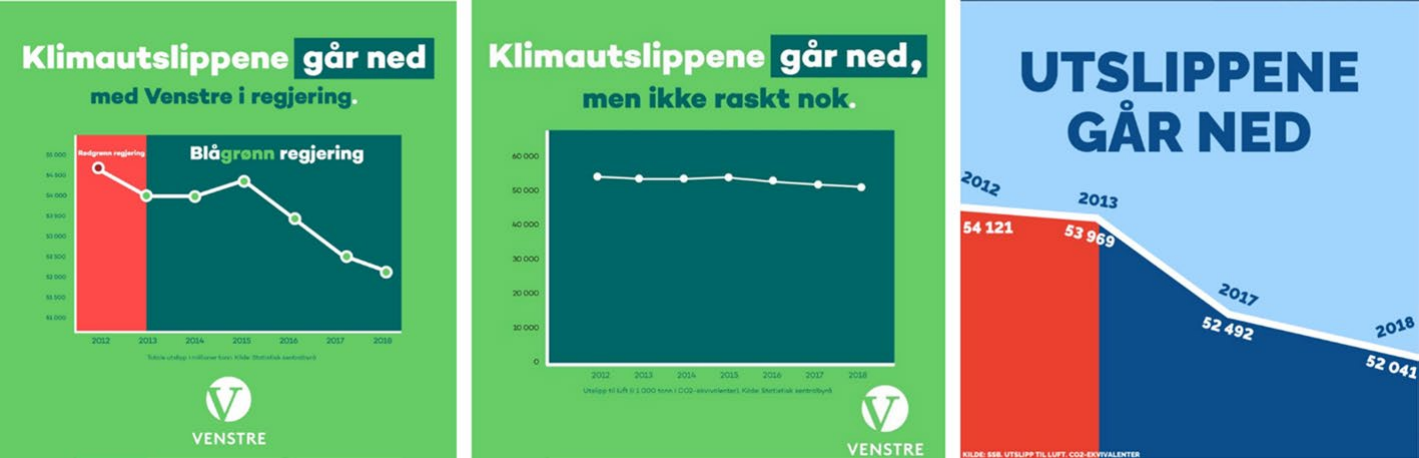
NRK (2019) retrieved the first two graphs from Venstre’s website, and Faktisk.no (2019) retrieved the third from Høyre’s website. The headings go, “Climate emissions are declining—with Venstre in the government”, “Climate emissions are declining, but not fast enough”, and “The emissions are declining”. The red area in the first and third graph represents the period previous to the government coalition where both Venstre and Høyre were joining. The first graph was replaced by the second after considerable criticism in the media for conveying a deceptive message

A graph showing the development of emission of greenhouse gases in Norway hit the news in 2019. Venstre (Norway’s Social Liberal Party), who had posted the graph on their website, was accused of greenwashing due to the choice of values on the vertical axis (NRK 2019). As a consequence, the graph was replaced. Figure 1 shows three graphs: The first on the left is the criticised graph, where the numbers of the axis range from 50,000 to 55,000, while in the replaced graph, the second one, they range from 0 to 60,000. At that time, Minister of Climate and Environment, representing Venstre, claimed both graphs to be correct (NRK 2019). The numbers in the graph may well be correct, but this example illustrates what Tufte (2001) calls deceptive use of graphic representation and what Mehta and Guzmán (2018) call spatial manipulation, where the choice of representation makes it look as if the emissions have declined drastically. This choice was probably intentional, since the red and dark green part of the first graph shows emissions during two government periods. This is not shown in the second graph, which makes sense, since it now seems to be little difference between the two governments.

The website of Høyre (Conservative Party of Norway), which was the lead government party at the time, was also scrutinised by the media. As the third graph in Fig. 1. shows, Høyre chose a similar presentation to Venstre, but in addition excluded the points that show an increase from year 2013 (Faktisk.no 2019). The daily newspaper Dagbladet (2019) called this cheating. If taken literally, the numbers might not be wrong, but the graph suggests that the numbers from 2014, 2015 and 2016 lie on the straight line between 2013 and 2017. This graph can thus be considered as more manipulative than the first graph from Venstre. Both graphs can serve as examples of *tweaked numbers*. They are deceptive as they convey a message of the government having successfully decreased emission levels. At the same time, they are less serious examples than *distorted numbers,* because it is relatively easy to discover that the actual development is rather insignificant.

The last example is taken from a discussion among master students in mathematics teacher education, where the lecturer presented a graph on predicted temperature changes, provided by the Intergovernmental Panel on Climate Change (IPCC) (see Hauge and Barwell 2017; Hauge 2016). The graph was constructed from a range of climate models, which all had produced predictions in accordance with a set of future scenarios. Some models were not represented after a certain year, and the graph made a drop in temperature predictions in all scenarios this specific year. IPCC reports are consensus based, but without requiring the applied mathematical models to produce exactly the same results. One of the students pointed to the graph, exclaiming that the results from the most pessimistic models were removed the specific year, suggesting that the remaining models might give a too optimistic picture (see Hauge and Barwell 2017; Hauge 2016). The student thus made the others aware of what may be categorised as a *bias*, assuming that there was no intention of masking pessimistic predictions. Because climate systems are complex, it cannot be expected that climate models provide equal predictions. This does not mean that predictions are not useful, so that IPCC predictions may also be characterised as *valid*. The questions in Table 1 can be useful for discussing why (or why not) numbers from IPCC can be trusted.

## Summary

This paper provides a set of questions to help reflecting on the validity of numbers or other mathematical representations, combined with whether they have a manipulative role in the context they are presented (see Table 1 and 2). The questions cover five areas: the numbers’ context, the communication form of the text, the numbers’ associated uncertainty, the source of information and reflexivity. The purpose of the questions and the categories is to provide a tool for critiquing and reflecting on the validity of numbers and their role in the given context. The questions and categories do not divide situations in clear groupings. Rather, they indicate qualities of numbers and their role. The presented examples illustrate various qualities and categories, underlining the point that there may not be an unequivocal choice of category. There are several reasons for this: First, there may not be sufficient information available to examine consensus or to evaluate the number and its role. Second, expert knowledge may be required, so that most people will not have sufficient capacity. Third, evaluating a manipulative role or the degree of uncertainty may not be a value free exercise, so that the conclusion may depend on perceived importance and stakes of the issue. Fourth, trust in the source of information, and how much the topic at hand is in harmony with own convictions, may play a considerable role. Fifth, any combination of the previous three reasons is possible. This implies that developing a set of unambiguous categories is not achievable.

Questions on context, communication, uncertainty, source and reflexivity on own capacity are relevant in any exercise when statistics or mathematical modelling is applied. As the questions and categories in Tables 1 and 2 also include questions related to a possible hidden agenda, they complement existing research on critical mathematics education. They are a supplement to Niss’ (2015) notion of validation because a possible manipulative role is highlighted, which is relevant when facing fake news or any kind of information where numbers or mathematical representations are used with the purpose of deceiving. This deceptive role also adds a dimension to Skovsmose’s (1994) *formatting power of mathematics:* In addition to influencing how we understand reality, mathematics may contribute to a deceptive understanding of reality. This manipulative perspective is also essential in developing critical citizenship through mathematics, an aspect adding to the existing literature on disinformation and education.

In their literature review on links between mathematics education and democracy, Sánchez Aguilar and Molina Zavaleta (2012) summarized their findings into key links. One was on practices where applied mathematics in society were critically reflected upon in classrooms, of which working with the role of numbers in public debates or in deceptive information would be an example. This requires that students are critically engaged with problems in real contexts, making knowledge meaningful, aspects Giroux (2018) and Chinn et al. (2021) have argued are essential in the age of fake news. It further requires that students learn that mathematics applied in society seldom gives either a correct or a wrong answer, an attribute that even small children can acknowledge when counting beans or animals at home (see Andersson and Wagner 2018). The categories with associated questions offered in the present paper can help illuminating various roles numbers have in such real-world problems. A key message is that getting acquainted with all categories is necessary to avoid a true—false dichotomy when considering numbers or mathematics based claims as fake news. Yet, attention should be given to worries about critical mathematics education contributing to a blind dismissal of numbers and science, observed in society (Marcone et al. 2019). Kollosche’s (2020) suggestion to introduce styles of reasoning in education to gain faith in factual reasoning may be one of many approaches. This is an interesting, complicated and important discussion to pursue in future research.

## Data Availability

Not applicable.

## Footnotes

1 https://www.buzzfeednews.com/article/janelytvynenko/coronavirus-fake-news-disinformation-rumors-hoaxes or www.faktisk.no.

2 See e.g. https://www.buzzfeednews.com/article/janelytvynenko/coronavirus-fake-news-disinformation-rumors-hoaxes or www.faktisk.no.
